# Cause-Effect Relationship of Varying Bonding Thicknesses in Dentin Adhesion of Universal Adhesives

**DOI:** 10.3290/j.jad.b3240695

**Published:** 2022-08-18

**Authors:** Arefin Alam, Abu Faem Mohammad Almas Chowdhury, Monica Yamauti, Pipop Saikaew, Shuhei Hoshika, Ricardo Marins Carvalho, Hidehiko Sano, Sharanbir K. Sidhu

**Affiliations:** a PhD Candidate, Department of Restorative Dentistry, Division of Oral Health Science, Hokkaido University Graduate School of Dental Medicine, Sapporo, Japan. Experimental procedure, data analysis, wrote manuscript.; b Associate Professor, Department of Conservative Dentistry and Endodontics, Sapporo Dental College and Hospital, Dhaka, Bangladesh. Experimental procedure, experimental design, co-wrote manuscript, discussed the results and commented on the manuscript at all stages.; c Associate Professor, Department of Restorative Dentistry, Division of Oral Health Science, Hokkaido University Graduate School of Dental Medicine, Sapporo, Japan. Experimental procedure, discussed the results and commented on the manuscript at all stages.; d Assistant Professor, Department of Operative Dentistry and Endodontics, Faculty of Dentistry, Mahidol University, Bangkok, Thailand. Contributed to discussion.; e Assistant Professor, Department of Restorative Dentistry, Division of Oral Health Science, Hokkaido University Graduate School of Dental Medicine, Sapporo, Japan. Contributed to discussion.; f Professor, Department of Oral Biological and Medical Sciences, Division of Biomaterials, University of British Columbia, Faculty of Dentistry, Vancouver, Canada. Idea, experimental design.; g Professor, Department of Restorative Dentistry, Division of Oral Health Science, Hokkaido University Graduate School of Dental Medicine, Sapporo, Japan. Idea, hypothesis, experimental design.; h Reader in Restorative Dentistry, Centre for Oral Bioengineering, Institute of Dentistry, Barts and The London School of Medicine and Dentistry, Queen Mary University of London, London, UK. Contributed to discussion, proofread manuscript.

**Keywords:** dentin, universal adhesives, additional coating, adhesive thickness, microtensile bond strength, mechanical properties.

## Abstract

**Purpose:**

To evaluate whether varying thicknesses of universal adhesives utilizing the additional coating strategy would affect their microtensile bond strength (µTBS) to dentin, hardness, and elastic modulus (mechanical properties).

**Materials and Methods:**

Ninety-nine human maxillary premolars were cut to expose coronal dentin, ground with regular-grit diamond burs, and randomly distributed into nine groups based on 1. adhesive: Scotchbond Universal Adhesive (SB; universal), G Premio Bond (GP; universal) and Clearfil Megabond 2 (MB; two-step self-etch; control); and 2. application strategy (one, two or three coats; each coat light cured). After adhesive application and resin composite buildup, the bonded teeth were stored in distilled water (37°C; 24 h). Resin-dentin sticks from eight premolars per group (each premolar yielded three sticks; n = 24 sticks altogether) were prepared for the µTBS test, followed by measurement of the adhesive thicknesses at their fractured ends using SEM. The mechanical properties of the adhesive layers produced by different coats were evaluated on separate resin-dentin slices (n = 3 teeth per group).

**Results:**

Two coats significantly increased the µTBS (p < 0.001) of all the adhesives. The correlation between adhesive thickness and bond strength was positive for GP but negative for SB. MB did not show any correlation. Additional coating significantly increased the mechanical properties of GP (p < 0.05) but did not affect SB and MB (p < 0.05).

**Conclusion:**

An additional adhesive coating over the manufacturers’ recommendations improved the bond strength of all the adhesives tested. However, the increased mechanical properties of the adhesives with additional curing was material dependent.

Since 2011, manufacturers have been promoting multi-mode adhesives, known as universal adhesives.^25^ As the name implies, universal adhesives can be used in either of the two application modes: etch-and-rinse or self-etch modes.^39^ They are designed similarly to the already existing “all-in-one” concept utilized in the “one-step self-etch” adhesives, continuing the trend of shortened application times compared to other adhesives,^26^ but with the possibility of being modified for more versatile indications.^3,39^ Notwithstanding, the one-step self-etch approach is applicable only on dentin, as it was found to be insufficient for enamel adhesion, unless the enamel is pre-etched with phosphoric acid, a procedure known as selective enamel etching.^5,16^

Water is an integral component of universal adhesives, making them more hydrophilic than two-bottle adhesives.^3^ Their higher hydrophilicity results in lower dentin bond strengths compared to two-step adhesives, an outcome similar to their predecessor – the one-step adhesives.^9,40^ Despite such limitations, the multi-functionality, reduced application time, and user friendliness of universal adhesives have maintained their demand and increased their use among clinicians. Studies have aimed to improve their bonding outcome to dentin by employing different clinically relevant approaches, such as covering the adhesive layer with an extra hydrophobic resin layer or enhancing adhesive application.^2,12^

Adhesive application can be enhanced either by applying additional layers and curing after each application (additional coating) or by additional applications but curing only at the end of the application procedure (increased application time).^10,34^ A thicker adhesive layer (additional coating), which exerts a cushioning effect against stress, also reduces the oxygen inhibition effects, leading to better polymerization.^6,29^ In contrast, increased application time can improve the bond strength by better resin infiltration and decreasing residual water; however, the latter effect is material dependent.^6,8^

Longer adhesive application time may promote adhesive pooling, resulting in a non-homogeneous and poorly polymerized adhesive layer.^41^ In contrast, the additional coating strategy can produce a more uniform adhesive layer.^21^ Moreover, additional light curing may also improve the polymerization via increased monomer conversion.^15^ Double coats or an extra hydrophobic resin layer have been found to improve the bonding efficacy of the universal adhesives.^2,12^ According to the findings of a recent study, a new two-step universal adhesive, G2 Bond Universal (a successor of G-Premio Bond), showed an adhesive thickness as high as 38 µm, with a bonding performance comparable to that of the gold standard two-step self-etch adhesive Clearfil Megabond 2 (22 µm).^11^

Nonetheless, if an adhesive layer is excessively thick, it would predominantly fail cohesively while bearing the functional load of a restoration due to the concentration of stress inside it.^35^ Moreover, a thick adhesive layer could be esthetically unacceptable at the restoration margins or appear as a caries-like radiolucent area in radiographs,^20^ unless the adhesive itself is radiopaque.^1^ Furthermore, to what extent the increased thickness of a universal adhesive could improve its dentin bond strength or other mechanical properties has not been established by a direct cause-effect relationship utilizing the same specimens to evaluate both variables. Therefore, it is crucial to investigate the effects of additional coating of current universal adhesives on their thickness, mechanical properties, and bond strength.

The aim of this study was to determine the correlation between the dentin bond strength of universal adhesives with their corresponding adhesive thickness when applied using an additional coating strategy. In addition, the effects of the additional curing on the mechanical properties of the adhesive layer were also determined. The null hypotheses tested were that the additional coating strategy would not: 1. improve the dentin bond strength and 2. enhance the mechanical properties of the universal adhesives.

## MATERIALS AND METHODS

### Tooth Selection and Specimen Preparation

A total of 99 sound human maxillary premolars were used in this study.^4^ The teeth were obtained with the patients’ informed consent and approved by the local Ethics Committee (protocol # 2018-9). All the teeth were cleaned and stored in a 0.5% aqueous chloramine-T solution at 4°C and used six months post-extraction.

The dental enamel was removed to expose the coronal dentin with five unidirectional gentle strokes of tapered regular-grit (63 µm) diamond burs (diamond point FG, #102R, Shofu; Kyoto, Japan) in a high-speed handpiece with adequate water cooling to simulate clinically relevant smear layers.^4,32^ Each bur was discarded after the preparation of five teeth. The teeth were randomly assigned to nine experimental groups to produce samples based on 1. adhesives: Scotchbond Universal Adhesive (SB, 3M Oral Care; St Paul, MN, USA, Universal) G-Premio Bond (GP, GC; Tokyo, Japan, Universal), and Clearfil Megabond 2 (MB, Kuraray Noritake, two-step, control); and 2. the number of adhesive coatings – one coat (according to the manufacturers’ instructions), two coats, or three coats (Table 1).

**Table 1:** Adhesives, their composition and application strategies

Adhesive (code/ batch no.)	Composition	^¶^Application strategies		
		*One coat	Two coats	Three coats
Scotchbond Universal Adhesive (SB/ 666963)	10-MDP, Vitrebond copolymer, HEMA, dimethacrylate resins, filler, silane, initiators, ethanol, water	Apply adhesive and rub for 20 s. Gently dry for about 5 s until it no longer moves, and the solvent evaporates. Light cure for 10 s.	Apply the first layer following steps 1–3 of the one-coat strategy. Apply the second layer and leave for 20 s. Repeat steps 2–3 of the one-coat strategy.	Apply two layers, following steps 1–3 of the two-coat strategy. Apply the third layer and leave for 20 s. Repeat steps 2–3 of the one-coat strategy.
G-Premio Bond (GP/ 1807031)	10-MDP, 4-META, MDTP, methacrylate acid ester, distilled water, acetone, photoinitiators, fine powdered silica	Apply adhesive and leave undisturbed for 10 s. Dry thoroughly with maximum air pressure. Light cure for 10 s.	Apply the first layer following steps 1–3 of the one-coat strategy. Apply the second layer and leave for 20 s. Repeat steps 2-3 of the one-coat strategy.	Apply two layers, following steps 1–3 of the two-coat strategy. Apply the third layer and leave for 20 s. Repeat steps 2–3 of the one-coat strategy.
Clearfil Megabond 2 (MB/ Japan/ 000047)	Primer: 10-MDP, HEMA, hydrophilic aliphatic dimethacrylate, dl-CQ, water. Bond: 10-MDP, bis-GMA, HEMA, dl-CQ, hydrophobic aliphatic dimethacrylate, initiators, accelerators, silanated colloidal silica.	Apply primer and leave for 20 s. Gently air blow for > 5 s. Apply bond. Gently air blow to make a uniform film. Light cure for 10 s.	Apply the first layer following steps 1–5 of the one-coat strategy. Apply the second layer repeating steps 3–5 of the one-coat strategy.	Apply the first layer, following steps 1–5 of the one-coat strategy. Apply the second and third layers by repeating steps 3–5 of the one-coat strategy twice.

^¶^Self-etch mode; *Manufacturer’s instruction. 10-MDP: 10-methacryloyloxydecyl dihydrogen phosphate; HEMA: 2-hydroxyethyl methacrylate; 4-META: 4-methacryloxyethyl trimellitic anhydride; MDTP: 10-methacryloyloxydecyl dihydrogen thiophosphate; CQ: camphorquinone; bis-GMA: bisphenol-A-glycidyl methacrylate.

Adhesive application was followed by building up three increments of resin composite (Clearfil AP-X, Kuraray Noritake), with each increment not exceeding 1.5 mm. Each adhesive coat was cured for 10 s (Table 1) and each resin composite increment was cured for 20 s with an LED curing unit (Pencure 2000, J Morita; Tokyo, Japan) having a power output (irradiance) of 1000 mW/cm^2^. The irradiance of the curing unit was checked periodically (Radiometer 100, Demetron Kerr; Orange, CA, USA). The bonded teeth were then stored in distilled water at 37°C for 24 h.

### Microtensile Bond Strength (µTBS) Test

Resin-dentin sticks (approximately 1 mm^2^ cross-sectional area) were prepared from 72 bonded teeth (eight teeth per group) with a low-speed diamond saw (IsoMet 1000, Buehler; Lake Bluff, IL, USA) according to the non-trimming technique.^4^ Three sticks per tooth (n = 24 sticks per group) were selected for testing. Each stick was attached to Ciucchi’s jig with cyanoacrylate glue (Model Repair II Blue, Dentsply-Sankin; Tokyo, Japan) and stressed under tension using a 500-N load cell at 1 mm/min crosshead speed in a desktop testing apparatus (EZ-S, Shimadzu; Kyoto, Japan) until failure occurred. The maximum load at failure was recorded. The data retrieved in N were divided by the cross-sectional area (mm^2^) to calculate the bond strength and expressed in MPa.

### Failure Mode Analysis

Failure modes were determined immediately after the µTBS test from observation of the sticks’ fractured ends using a stereomicroscope at 50X magnification (SMZ-171-TLED, Shimadzu), ensuring that the specimens were not dehydrated.^4^ The failure modes were classified as adhesive failure, cohesive failure in dentin, cohesive failure in resin composite, and mixed failure.^14^ For the sake of simplification, these four modes were reclassified into adhesive failure and non-adhesive failure.^23^ The failures occurring individually or simultaneously at the resin composite-adhesive interface, adhesive-dentin interface, or cohesively within the adhesive were considered adhesive failures. The non-adhesive failures included cohesive failure in dentin, cohesive failure in resin composite, and mixed failure.

### Measurement of Adhesive Thickness from Fractured Resin-Dentin Sticks

**Fig 1:**
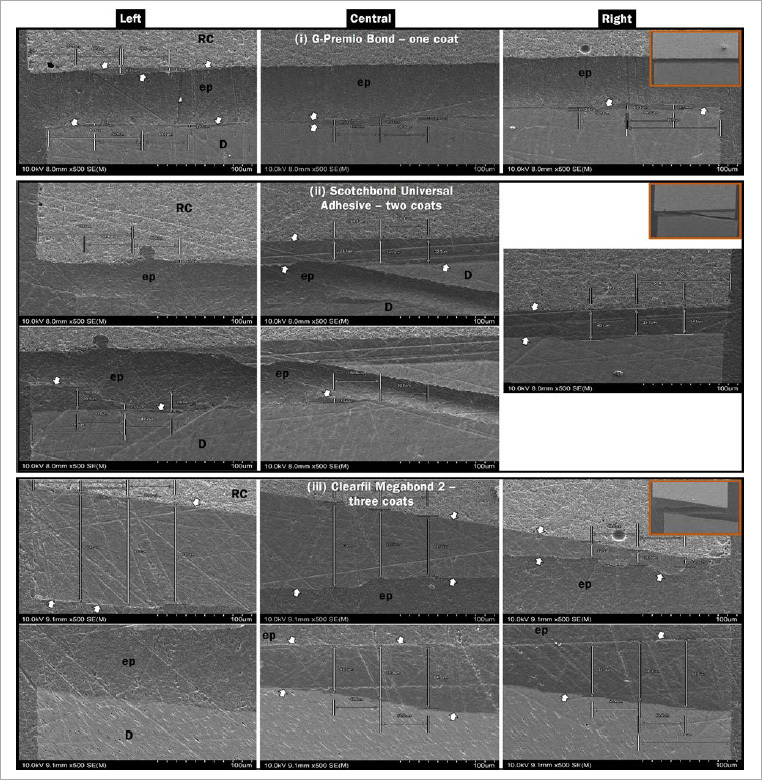
Representative SEM images showing the measuring locations of the adhesive layers from the fractured resin-dentin pairs of (i) G-Premio Bond applied in one coat, (ii) Scotchbond Universal Adhesive applied in two coats, and (iii) Clearfil Megabond 2 applied in three coats. Note that whenever close alignment of the fractured pairs was not achievable (ii and iii), the adhesive thicknesses of two corresponding sides could not be measured under the same focus leading to two separate images. The orange-bordered small-scale images show the complete widths of the fractured pairs. White arrows indicate the adhesive layers; ep: epoxy resin; D: dentin; RC: resin composite.

The adhesive layer thickness was measured using a scanning electron microscope (SEM, FE-SEM, S-4800, Hitachi; Tokyo, Japan). The measurement was done from both ends of the fractured resin-dentin sticks immediately after failure mode analysis (Fig 1).

Flat plastic rings (diameter = 6 mm; height = 1 mm) were fixed over aluminum stubs. The same surfaces of each pair of fractured fragments were demarcated to align the fractured ends of the resin-dentin sticks properly inside the rings. After epoxy embedment, the specimens were sequentially wet-polished with SiC papers (#600, #800, and #1000 grit, Sankyo-Rikagaku; Saitama, Japan) and diamond pastes (6, 3, and 1 µm, DP-Paste, Struers; Ballerup, Denmark). Ultrasonic cleaning was done for 2 min after each polishing step. The specimens were then dried at room temperature (23 ± 2°C; 50 ± 5% RH) for 24 h, sputter-coated with Pt-Pd (E-1030, Hitachi, Tokyo, Japan), and observed under the FE-SEM at 500X magnification at an accelerating voltage of 10 kV to measure the adhesive thickness. The thickness was measured with the built-in scaling tool of the SEM image processing software. As shown in Fig 1, the adhesive thickness (µm) was calculated at three different locations from each fractured end. The locations were at the left lateral, right lateral, and central areas of each end. Each lateral location was approximately 100 µm medial to the respective edge of a fractured end, and the central spot was located approximately halfway between the edges. The value at each location corresponded to the mean of triplicate measurements. The average thickness value of all three spots of one fractured end was considered as its adhesive thickness. Finally, the sum total value of each stick’s fractured-end adhesive thickness was considered the approximate adhesive thickness of that stick.

### Evaluation of Hardness and Elastic Modulus of the Adhesive

**Fig 2:**
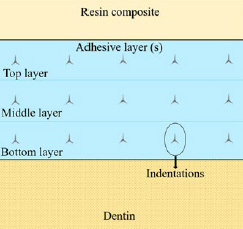
Schematic of the indentation test for measuring the hardness and elastic modulus of the adhesive layer(s).

After 24 h of water storage (37°C), 27 similarly bonded teeth (n = 3/group) were longitudinally sectioned with an IsoMet saw (Buehler) to form 1.5-mm-thick resin-dentin slices. One central slice per tooth was used for hardness and elastic modulus evaluation. Each slice was sequentially polished with wet SiC paper of decreasing abrasiveness (#1000, #1200, and #2000 grit) followed by diamond paste polishing of up to 1-µm grain. Each polishing step followed ultrasonic cleaning with distilled water for at least 1 min. The hardness and elastic modulus of the adhesive layer was measured with a dynamic ultra-microhardness tester (DUH-211, Shimadzu) having a 0.1 µm Berkovich diamond indenter with a 115-degree angulated tip (ambient conditions: 23 ± 2°C and 50 ± 5% RH). The procedure was conducted by indenting at five different spots/adhesive coat/slice at an interval of approximately 200 µm. Indentations at excessively thinned adhesive regions were avoided. The indentations were performed at a constant speed of 0.2926 mN/s. The maximum load employed was 5.04 mN with a 10-s holding time, and setting Poisson’s ratio at 0.30.^13^ A clear distinction between two adhesive layers of all three adhesives was visualized with the built-in microscope of the testing device, permitting indentation placement approximately in the mid-thickness of each layer. The two-coat groups received indentations at the bottom of the adhesive layer (produced by the first coat) as well as at the top adhesive layer (produced by the last coat). Likewise, the three-coat groups received indentations at the bottom, middle, and top adhesive layers (Fig 2). Finally, each adhesive layer’s mean hardness (MPa) and elastic modulus (GPa) were obtained. In the case of two or three coats, mean values of the multiple coats were also calculated.

### Statistical Analysis

Data were analyzed using SPSS 24.0 (Chicago, IL, USA) with significance set at α = 0.05. The µTBS data were distributed normally and homogeneously. Two-way ANOVA was done to check the effects of the adhesives and their number of coats on µTBS. The statistical differences between the nine study groups were further checked with one-way ANOVA and Tukey’s post-hoc test. Adhesive thickness, hardness, and elastic modulus data were non-normal and non-homogeneous. Therefore, a Kruskal-Wallis H-test, followed by Dunn’s post-hoc test adjusted with Bonferroni correction, was performed to demonstrate the effects of different coats of adhesives on their thicknesses and mechanical properties. The resin-dentin stick was considered the statistical unit, and Spearman’s rank-order correlation test was done to correlate the µTBS with the same stick’s adhesive thickness. For analyzing the hardness and elastic modulus of the adhesive layers in multiple-coat groups, the Mann-Whitney U-test (two-coat) and Kruskal-Wallis H-test with pairwise comparisons (three-coat) were performed.

## RESULTS

### µTBS

**Fig 3:**
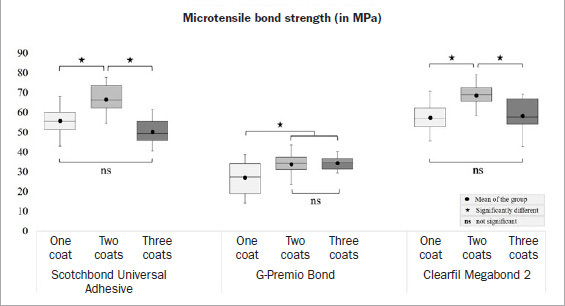
Box-and-whisker plot (minimum-(lower quartile-median-upper quartile)-maximum) of microtensile bond strengths obtained by the adhesives applied in different numbers of coats.

There were no pre-test failures. Two-way ANOVA revealed significant effects of adhesives (F = 476.263, p < 0.001) and number of coats (F = 51.625, p < 0.001) on the µTBS. The interaction between these variables were also significant (F = 12.498, p < 0.001). The µTBS test result is graphically represented in Fig 3. Regardless of the adhesives, the application of two coats resulted in significantly higher µTBS than their one-coat counterparts (p < 0.05). However, in the three-coat groups, the µTBS of SB and MB decreased significantly compared to their corresponding two-coat groups (p < 0.05) but were similar to their one-coat groups (p > 0.05). In contrast, the µTBS of the three-coat group for GP was not significantly higher than its two-coat counterpart (p > 0.05).

### Failure Modes

**Fig 4:**
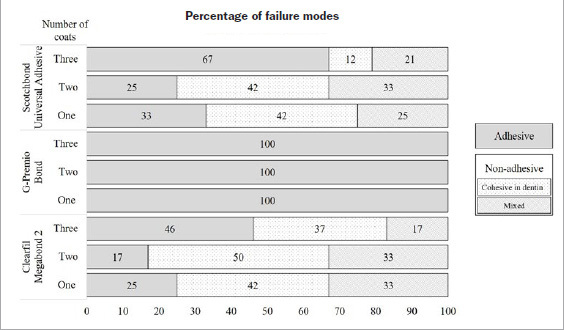
Fracture modes of the tested adhesives applied in one, two or three coats. The failures occurring individually or simultaneously at the resin composite-adhesive interface, adhesive-dentin interface, and cohesively within the adhesive were considered adhesive failures. The non-adhesive failures include cohesive failures in dentin or mixed failure types involving dentin. Cohesive failure in resin composite was not observed.

Figure 4 shows the percentage of failure modes. Irrespective of the number of coats, non-adhesive failures were predominant in SB and MB, except for the three-coat group of SB, which mainly showed adhesive failures. All the GP specimens demonstrated adhesive failures only. No cohesive failure in resin composite was observed in the study.

### Adhesive Thickness

**Fig 5:**
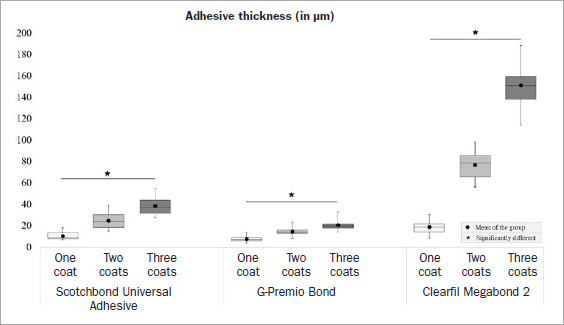
Box-and-whisker plot (minimum-(lower quartile-median-upper quartile)-maximum) of adhesive thicknesses obtained by the adhesives applied in different coats and measured from the fractured resin-dentin sticks following the microtensile bond strength test.

The adhesive thickness results are summarized in Fig 5. The increased number of coats resulted in a significant increase in adhesive thicknesses for all adhesives (p < 0.001). The thickest adhesive layer among the same coating groups was observed in MB, followed by SB and GP, regardless of the number of coats. Figure 1 shows representative SEM images of the adhesive thickness measured on the fractured interfaces, where the GP one-coat group had the thinnest, the SB two-coat group had somewhat thicker, and the MB three-coat group had the thickest adhesive layers.

**Fig 6:**
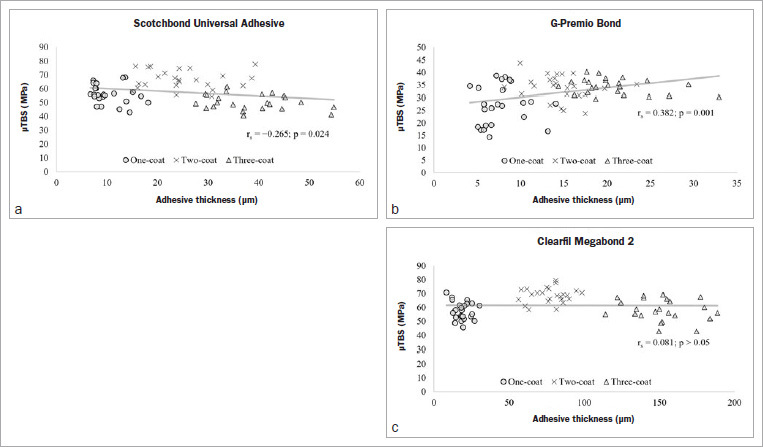
Correlation between adhesive thickness and corresponding microtensile bond strength (µTBS) of the tested adhesives applied in one, two, and three coats.

**Fig 7:**
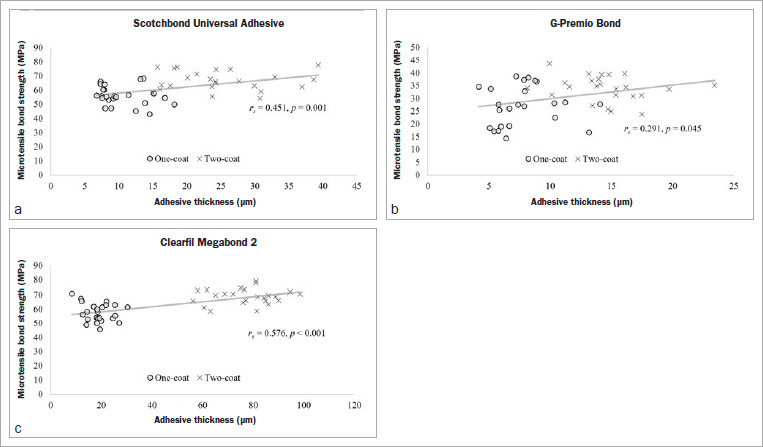
Correlation between adhesive thickness and corresponding microtensile bond strength (*µ*TBS) of the adhesives applied in one and two coats.

As shown in Fig 6, SB and GP both exhibited significant correlations between bond strength and adhesive thickness (p < 0.05), but in contrasting coefficients, with SB being weak and negative (r_s_ = -0.265) and GP being moderately positive (r_s_ = 0.382). MB did not show any correlation (r_s_ = 0.089, p > 0.456). However, the correlations for all three adhesives were weak to moderately positive and significant until application in two coats (Fig 7): SB (r_s_ = 0.451, p = 0.001), GP (r_s_ = 0.291, p = 0.045) and MB (r_s_ = 0.576, p < 0.001).

### Hardness and Elastic Modulus

**Fig 8:**
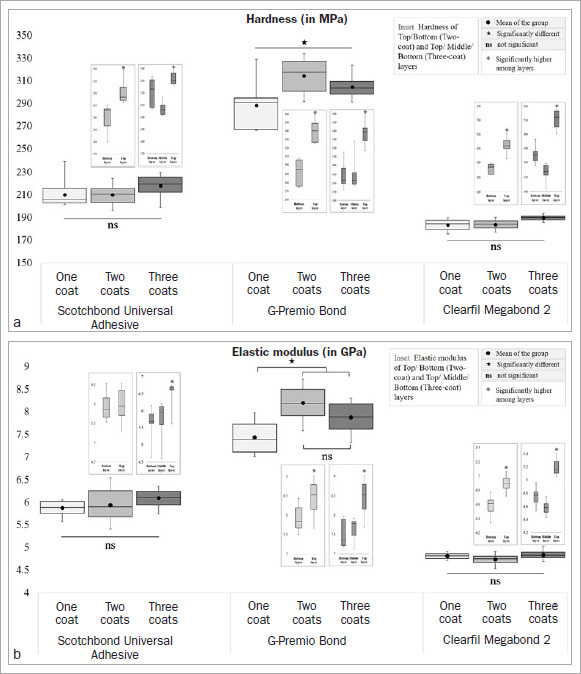
Box-and-whisker plot (minimum-(lower quartile- median-upper quartile)-maximum) of hardness (a) and elastic modulus (b) values obtained by the adhesives applied in different numbers of coats.

The hardness and elastic modulus values of the adhesive layer(s) obtained with different application strategies are illustrated in Fig 8. Multiple coats did not affect the mechanical properties of SB and MB (p > 0.05). For GP, additional coats significantly increased both the hardness and elastic modulus values compared to the one-coat counterpart (p < 0.05). As shown in the insets of Fig 8, the top adhesive layers (Fig 2) in the case of multiple-coat groups obtained the highest values (p < 0.05), except for the elastic modulus of SB’s two-coat group.

## DISCUSSION

The bond strength of adhesives applied in different thicknesses has been tested before, using either bonded dentin slices or resin-dentin sticks to measure the adhesive thickness.^10,34,41^ The dentin bond strengths of several adhesives have been found to benefit from application in thicker layers.^18,34^ Ausiello et al^6^ evaluated the effects of increased adhesive thickness by 3D finite-element analysis. They concluded that an optimal adhesive layer thickness could lead to maximum stress relief, improving the dentin bond strength. The results of the present study, which focused on obtaining the bond strengths and adhesive layer thicknesses of the same resin-dentin sticks, also agree with these reports. In our case, after the 24-h bond strength test, the fractured ends of the sticks were immediately subjected to failure mode analysis, and the adhesive layer thickness was measured using SEM. The evaluation method employed in our investigation allowed the determination of a direct and representative cause (adhesive thickness) and effect (bond strength) relationship by using the resin-dentin sticks for the µTBS test, followed by measurement of the adhesive thickness from the fractured ends of the same sticks with SEM.

This study revealed that, regardless of the adhesive, a second coat significantly increased the adhesive thickness and the bond strength (p < 0.05). In contrast, despite forming the thickest adhesive layer, a third coat did not improve the bond strength any further, but in fact decreased it. This finding was complemented by the weak but significant and inverse correlation for SB (r_s_ = -0.265) and no correlation for MB (r_s_ = 0.089, p = 0.456) (Fig 6a,c). Although GP showed a positive correlation (r_s_ = 0.382, p < 0.05) (Fig 6b), the mean bond strength of its three-coat group (38.9 ± 3.2 MPa) was not significantly different (p > 0.05) from its two-coat counterpart (37.2 ± 5.1 MPa) (Fig 3), indicating achievement of a plateau at two coats. Therefore, the first null hypothesis had to be partially rejected.

The significantly improved bonding performances of the SB and MB two-coat groups were substantiated by the increasing non-adhesive failure percentages (Fig 4). According to previous reports, the predominant failure in HEMA-free GP is adhesive-dentin interfacial fracture which occurs as a consequence of phase separation.^31,38^ Also, in this study, the failure pattern of GP was always adhesive, regardless of the adhesive thickness and bond strength. Moreover, one coat of GP produced the thinnest adhesive layer (7.8 ± 2.6 µm) among all the tested groups. Nevertheless, multiple light exposures radiating through the additional thin films in the case of GP’s two- or three-coat groups might have benefitted the bottom layer.^19,29^ Therefore, it seems that in all the tested adhesives, two-coat applications led to an optimum thickness at which the bond strength reached its peak (Fig 7). This observation is also in agreement with previous reports.^12,18,21,29^

The mechanical properties, ie, the hardness and elastic modulus of an adhesive, can modify the bond strength by influencing the fracture resistance of the adhesive.^35^ For instance, lower mechanical properties increase the chances of adhesive failure.^7,35^ We hypothezised that the additional curing steps would enhance the mechanical properties of the adhesive layer(s), leading to improved µTBS. Therefore, we evaluated the hardness and elastic modulus values of the different adhesive layers (bottom, middle, and top) as an indirect indicator of the degree of conversion.^30^ However, the tested groups’ hardness and elastic modulus values (Fig 8) showed material dependency; GP demonstrated a significant increase in both properties with additional curing, whereas SB and MB did not show any significant change (p > 0.05). Thus, the second null hypothesis was also partially rejected. Taschner et al^36^ reported sufficient curing capability with a high degree of conversion of SB and concluded that additional coating would not improve it any further. Similarly, Clearfil Megabond 2, the improved version of Clearfil Megabond, contains an additional photoinitiator and a new accelerator, both of which have been claimed to be responsible for a high degree of conversion.^33^ In contrast, GP, being thin enough to suffer from oxygen inhibition, may have benefitted from the additional coating, resulting in increased mechanical properties.^27,37^

Interestingly, regardless of the adhesive, with an additional coating, the top adhesive layers demonstrated significantly higher hardness and elastic modulus values (p < 0.05) compared to the bottom layers (Fig 8), except for the elastic modulus of the two-coat of SB. This phenomenon may be a combined result of the following: firstly, the resin composite may have dislodged or absorbed some residual monomers from the underlying top adhesive layer, resulting in a better degree of conversion, and secondly, the heat generated during polymerization of the resin composite may also have resulted in improved monomer conversion at the adjacent adhesive layer.^29^

The present study results revealed that applying an additional coat (two-coat) was beneficial for all tested adhesives. In GP, the application of two coats improved both adhesive thickness and mechanical properties leading to increased µTBS. On the contrary, the bond strength improvement of SB and MB resulted from increased adhesive thickness only. Nevertheless, from a clinical perspective, a thicker adhesive layer created with an additional coat beneath a resin composite restoration may trigger esthetic or diagnostic concerns. The polymeric structure of fillers and the surface roughness of an adhesive can accumulate stains from various sources inside the oral cavity over time, leading to marginal discoloration.^7,17^ Referencing Jorgensen, Fusayama reported that the minimal visible space between the preparation margin and an inlay was 50 µm.^22^ Therefore, adhesive thicknesses below this limit may be esthetically tolerable. However, according to Opdam et al,^28^ an adhesive layer thicker than 40 µm would be detectable as a radiolucent area underneath the restoration. The addition of some degree of radiopacity to the current adhesives could avoid misdiagnosing them as caries. The new Scotchbond Universal Plus Adhesive, a successor of the SB, is claimed to contain a novel type of resin providing the adhesive layer with dentin-like radiopacity.^1^

While one additional adhesive coat is promising with improved immediate performance, the long-term bonding outcome would confirm the eligibility of such an application. An additional adhesive layer may contain non-neutralized acidic monomers and solvents, provoking internal plasticization of the layer itself, lowering the cohesive forces between the polymer molecules.^24^ Future studies involving such application strategies should consider thermocycling or prolonged water storage of the specimens.

## CONCLUSION

1. An additional adhesive coating over the manufacturer’s recommended adhesive layer improved the bond strength of all the adhesives tested.
2. While increased adhesive thickness resulting from additional coating favored all the adhesives’ bond strengths, the beneficial effect of additional curing was material dependent.

## Clinical relevance:

Application of universal adhesives in two coats can help them obtain the optimum thickness beneficial for bond strength.
